# *Salmonella enterica* serovar typhi limits the potency of typhoid toxin and ADP‐ribosylating toxin AB to establish a persistent infection

**DOI:** 10.1002/2211-5463.70329

**Published:** 2026-08-26

**Authors:** Maria C. Zabala‐Rodriguez, Juliette K. Tinker, Teresa Frisan, Taj Azarian, Ken Teter

**Affiliations:** ^1^ Burnett School of Biomedical Sciences University of Central Florida Orlando FL USA; ^2^ Department of Biology Boise State University ID USA; ^3^ Department of Molecular Biology and Umeå Centre for Microbial Research (UCMR) Umeå University Sweden

**Keywords:** AB toxins, ArtAB, balanced pathogenicity, Hsp90, pertussis‐like toxin, stealth infection, toxin translocation, typhoid toxin

## Abstract

Chronic infection is important for the transmission of *Salmonella enterica* serovar Typhi (*S*. Typhi), an obligate human pathogen responsible for typhoid fever. The immunomodulatory action of typhoid toxin helps establish an initial stealth invasion of the intestinal tract that can lead to systemic spread, colonization of the gallbladder, and persistent shedding. Typhoid toxin contains a cell‐binding pentamer and two catalytic subunits: cytolethal distending toxin B (CdtB, a DNase) and pertussis‐like toxin A (PltA, an ADP‐ribosyltransferase). It undergoes endocytosis and transport to the endoplasmic reticulum (ER) where toxin disassembly occurs. This allows the catalytic subunits to exit the ER for interaction with their nuclear or cytosolic targets. However, all known cellular and physiological effects of typhoid toxin are attributed to CdtB. These effects reduce the severity of intestinal inflammation, promoting host survival and long‐term infections. PltA has *in vitro* enzymatic activity but does not affect cells challenged with typhoid toxin. It is thought that the cytosolic action of PltA will be identified in future work. We instead propose that PltA never reaches the cytosol: it is retained in the ER to limit toxin potency, thereby allowing the anti‐inflammatory effects of CdtB to promote intestinal colonization. The absence of *Salmonella* ADP‐ribosylating toxin AB (ArtAB) from all strains of *S*. Typhi may serve a similar purpose. We accordingly propose a model of balanced pathogenicity in which the lack of toxin ADP‐ribosyltransferase activity is essential for *S*. Typhi pathogenesis.

## Graphical abstract

The two catalytic subunits of typhoid toxin dissociate from the holotoxin in the ER of an intoxicated cell, but only CdtB exits the ER to generate immunosuppressive effects. PltA is retained in the ER and sequestered from its cytosolic target, thus allowing the anti‐inflammatory effects of CdtB to promote intestinal colonization. Created in biorender, agreement #GK2A2HXOMW. Teter, K. (2026) https://biorender.com/ffaxf3z.

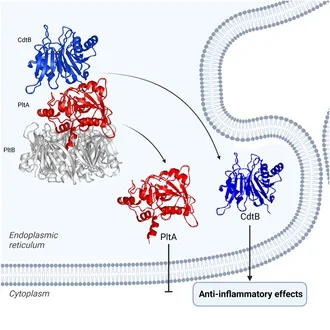

**Abbreviations**
ADPRT: ADP‐ribosylating toxin
ArtAB: *Salmonella* ADP‐ribosylating toxin AB
CdtB: cytolethal distending toxin B
CHO: Chinese hamster ovary
CT: cholera toxin
CTA1: catalytic A1 subunit of cholera toxin
ER: endoplasmic reticulum
NTS: nontyphoidal *Salmonella*
PBS: phosphate‐buffered saline
PltA: pertussis‐like toxin A
PltB: pertussis‐like toxin B
*S*. Typhi: *Salmonella enterica* serovar Typhi

*Salmonella enterica* serovar Typhi (*S*. Typhi), an intracellular pathogen responsible for ~ 200 000 annual deaths from typhoid fever, produces typhoid toxin [1]. Several nontyphoidal *Salmonella* (NTS) strains also produce the toxin [2], which contains a cell‐binding homopentamer of the pertussis‐like toxin B protein (PltB) and two distinct catalytic subunits: pertussis‐like toxin A (PltA) and cytolethal distending toxin B (CdtB). PltA is an ADP‐ribosylating toxin (ADPRT) that sits within and above the central cavity of the PltB pentamer. CdtB, a DNase, does not interact directly with PltB but is instead anchored to the toxin via a disulfide bridge with PltA (Fig. 1) [3, 4].

**Fig. 1:**
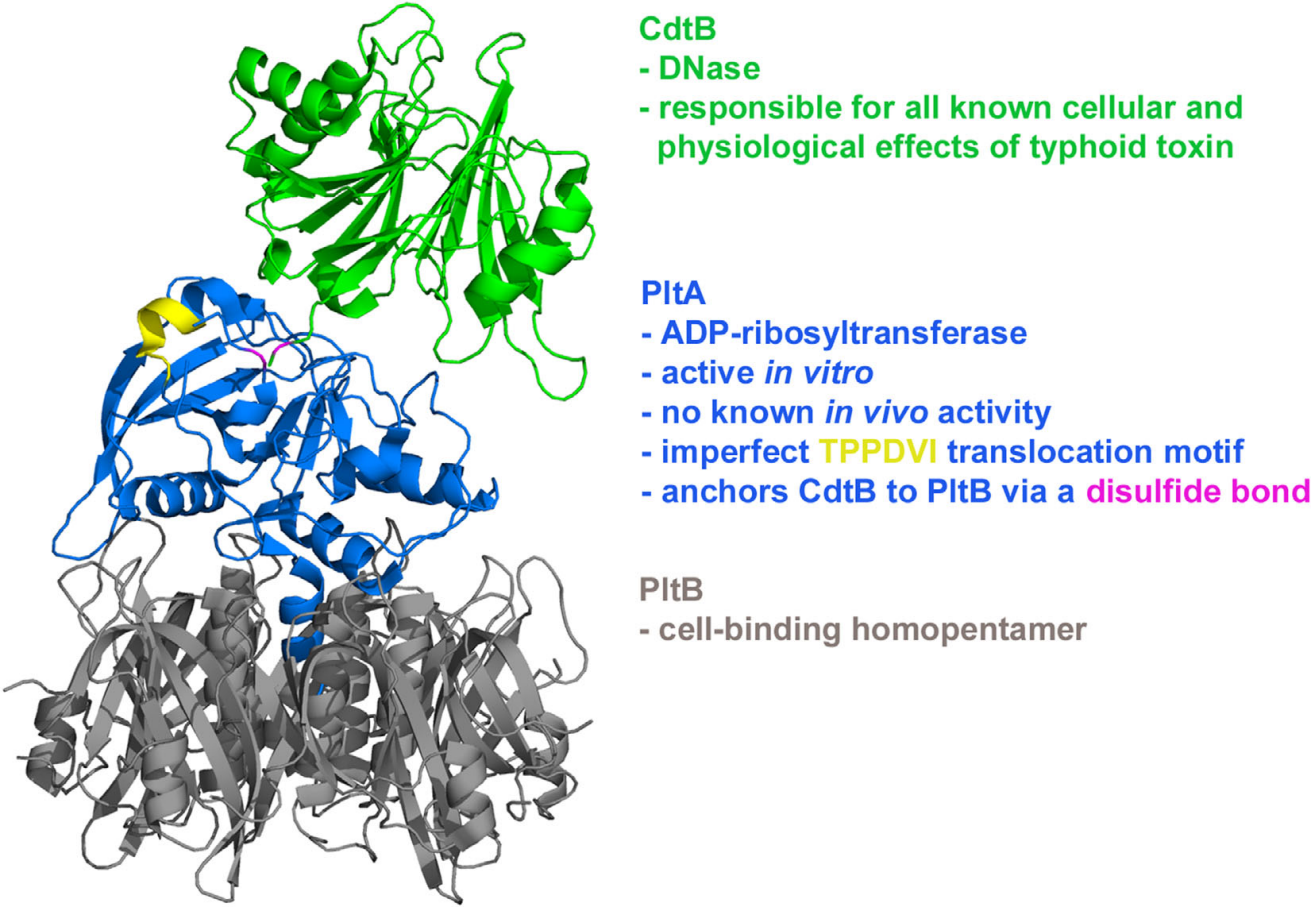
Structural organization of typhoid toxin. Typhoid toxin contains a DNase (CdtB, green), an ADP‐ribosyltransferase (PltA, blue), and a cell‐binding pentamer (PltB, gray). CdtB is linked to PltA by a disulfide bond (magenta), while non‐covalent contacts position PltA within and above the central cavity of the ring‐like PltB pentamer. In some cases, the cell‐binding subunit of ArtAB (ArtB, aka PltC) can serve as a cell‐binding component of typhoid toxin. PBD entry 4K6L [3]; pymol was used to generate the protein structure.

Typhoid fever begins with a bacterial invasion of the intestinal tract that involves an asymptomatic incubation period of 6–30 days. *S*. Typhi then causes a systemic infection with dissemination to distal sites and possible chronic colonization of the gallbladder, an infrequent (≤ 5% of cases) but important event for this obligate human pathogen [5, 6, 7]. The lack of an effective inflammatory response during the early stage of infection is due in part to the immunomodulatory properties of the CdtB subunit in typhoid toxin [8, 9, 10, 11, 12].

After binding to the plasma membrane of a susceptible cell, typhoid toxin undergoes receptor‐mediated endocytosis and retrograde vesicular transport to the endoplasmic reticulum (ER) [4, 13, 14]. Reduction of the CdtB/PltA disulfide bond in the ER releases CdtB from the rest of the toxin [3]. An interaction between the ER‐localized store of ATP and the PltB pentamer may produce a conformational change in PltB that dislodges PltA from the pentamer, similar to the process for pertussis toxin [15]. The free catalytic subunits can then enter the cytoplasm and nucleoplasm for a toxic effect. However, all known cellular and physiological effects of typhoid toxin are attributed to CdtB [3, 4, 16]. PltA has demonstrable enzymatic activity *in vitro*, but an ADP‐ribosylated protein in cells challenged with typhoid toxin has not been reported [4]. In fact, an inactive mutant of PltA does not affect the potency of typhoid toxin [3, 4]. PltA is required to anchor CdtB to the PltB pentamer [3, 4] (Fig. 1), but its direct enzymatic role in intoxication remains unknown. It is currently assumed that the function and cytosolic target of PltA will be identified in future work [2, 3, 9, 17, 18]. In contrast to this line of thought, we hypothesize that PltA activity in the cytosol would counteract the immunomodulatory effects of CdtB which prevent an intestinal inflammatory response [8, 9, 10, 11, 12]. The cytosolic delivery of PltA is therefore blocked so typhoid toxin can establish an initial, anti‐inflammatory stealth invasion of the intestines during the acute phase of infection. Below, we provide a molecular basis for the potential retention of PltA in the ER and note the absence of another *Salmonella* ADPRT from the *S*. Typhi genome. We then consider how the cytosolic activity of an ADPRT could disrupt the immunosuppressive effects of *S*. Typhi virulence factors such as CdtB and suggest experimental approaches to test the model.

## Results and discussion

### An imperfect binding motif for Hsp90 traps the toxin a chain in the ER

**Fig. 2:**
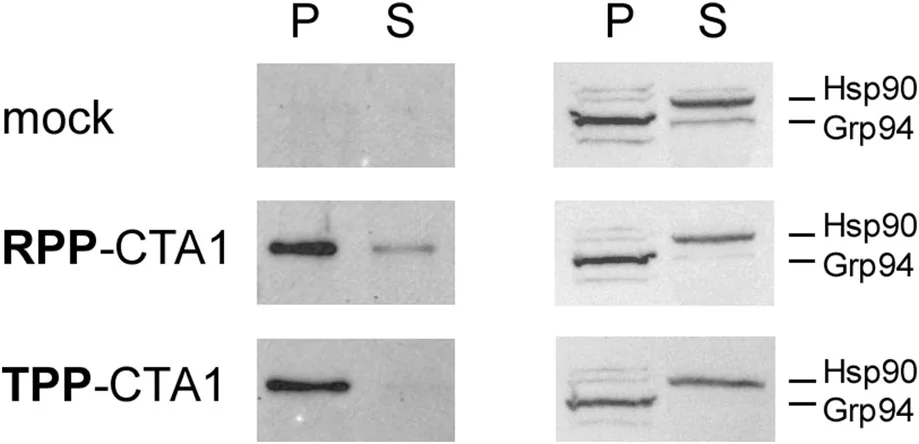
**TPPDEI**‐CTA1 is trapped in the endomembrane system. CHO cells were transfected with an empty vector (mock) or plasmids encoding ER‐localized variants of wild‐type **RPPDEI**‐CTA1 or mutant **TPPDEI**‐CTA1. Targeting to the ER was accomplished by appending the protein with an N‐terminal signal sequence that is cleaved in the ER to generate a mature toxin subunit that has previously been located in the ER by microscopy and biochemical methods [58]. The mature, ER‐localized toxin can then possibly undergo retrotranslocation back to the cytosol. At 24 h post‐transfection, digitonin‐permeabilized cells were partitioned into separate organelle (pellet, P) and cytosol (supernatant, S) fractions by centrifugation. (Left panel) Both fractions were probed by western blot for the distribution of CTA1. All images were taken from one exposure of a single membrane containing all samples. This result was originally reported in [20]; a new experiment (*n* = 2) is shown here to emphasize the reproducibility of the observation. The protein ran at a mobility between the 20 and 25 kDa molecular mass standards, which are not shown in the western blot image. (Right panel) For each transfection, western blots documented the partitioning of Grp94 (a protein in the ER lumen) to the P fraction and Hsp90 (a cytosolic protein) to the S fraction. A pilot experiment using separate blots for the Hsp90 and Grp94 antibodies confirmed that each antibody only recognizes its cognate protein and that Grp94 runs at a slightly faster mobility than Hsp90. Both proteins ran at a mobility near the 100 kDa molecular mass standards, which are not shown in the western blot image.

Several ER‐translocating toxins, including typhoid toxin, contain catalytic subunits (aka A chains) that modify their targets by ADP‐ribosylation. The toxin A chains spontaneously unfold after their ER‐localized dissociation from the cell‐binding component of the holotoxin and move through a “translocon” pore in the ER membrane to reach the cytosol [19]. Most, if not all, of these toxins rely on the cytosolic chaperone Hsp90 for passage into the cytosol [20, 21, 22, 23]. Using cholera toxin (CT) as a prototypical ER‐translocating ADPRT, we reported that Hsp90 couples toxin refolding with toxin extraction from the ER [24]. Hsp90 binds to the catalytic CTA1 subunit as it emerges at the cytosolic face of the ER membrane. Hsp90 then refolds the toxin, which prevents the cytosolic portion of CTA1 from sliding back into the narrow diameter of the translocon pore. The unidirectional movement of CTA1 from the ER to the cytosol thus occurs by a mechanism similar to the Brownian ratchet that is responsible for co‐translational protein import into the ER [25]. The extraction of CTA1 from the ER begins when Hsp90 recognizes an **RPPDEI** amino acid sequence near the N‐terminus of CTA1 (residues 11–16) [20]. The A chains from other ER‐translocating ADPRTs contain similar N‐ or C‐terminal sequences (Table 1), but the motif is absent from ER‐translocating toxins that are not ADPRTs [20, 26]. Alterations to any of the highly conserved **RPP** residues, including a **TPPDEI** mutation, blocked CTA1 passage from the endomembrane system to the cytosol [20] (Fig. 2). We believe this observation is key to understanding the pathogenesis of typhoid toxin.

**Table 1:** Distribution of the **RPP**‐based translocation motif in ER‐translocating ADPRTs. The A chains of most ER‐translocating ADPRTs contain an **RPP**‐based translocation motif near the N or C termini of the toxin. PltA from typhoid toxin is an exception to this observation. ER‐translocating toxins that are not ADPRTs lack an **RPP** motif, and only toxins with the motif exhibit a pattern of Hsp90‐dependent intoxication and translocation [26]. The amino acids comprising the **RPP** motif are numbered from the N‐terminus of the mature toxin subunit (i.e., with the signal sequence removed). For reference, there are 613 amino acids in exotoxin A.

ADPRT	Pathogen(s)	RPP‐like motif_(amino acid residues)_
Cholera toxin	*V. cholerae*	N‐terminal RPPDEI_(11–16)_
Pertussis toxin	*B. pertussis*	N‐terminal RPPEDV_(13–18)_
Exotoxin A	*P. aeruginosa*	C‐terminal PGKPPR_(604–609)_
Heat‐labile enterotoxin	*E. coli*	N‐terminal RPPDEI_(11–16)_
Pertussis‐like toxin	*E. coli*	N‐terminal RPPEEI_(10–15)_
ArtAB	NTS	N‐terminal RPPDVI_(10–15)_
Typhoid toxin, PltA subunit	NTS, *S.* Typhi and Paratyphi	N‐terminal TPPDVI_(10–15)_

We hypothesize that the **TPPDVI** sequence in PltA is not a substrate for Hsp90‐dependent translocation to the cytosol. PltA is thus trapped in the ER and cannot reach its cytosolic target. If PltA could enter the cytosol, the ADP‐ribosylation of this target would likely replace stealth intestinal invasion with an inflammatory infection that prevents asymptomatic carriage by supplanting the early immunosuppressive effects of CdtB and other *S*. Typhi virulence factors [27, 28]. Thus, typhoid toxin may have been driven to a state of balanced pathogenicity in which an **R**‐to‐**T** substitution in the **RPP** translocation motif limits its potency.

PltA is required for assembly of the typhoid holotoxin, serving as the physical bridge between CdtB and the PltB pentamer (Fig. 1) [3, 4]. Selection for a translocation‐defective variant would therefore prevent the cytosolic ADP‐ribosyltransferase activity of PltA while preserving toxin assembly, trafficking to the ER, and CdtB delivery to the nucleus. Such an adaptation would provide a parsimonious mechanism for retaining the anti‐inflammatory and persistence‐promoting properties of typhoid toxin while minimizing the detrimental consequences of PltA intoxication.

An active site mutation in PltA would eliminate its cytosolic activity, yet the toxin instead appears to contain a non‐functional **TPPDVI** translocation motif. The evolutionary selection for toxin sequestration in the ER rather than toxin inactivation remains unknown. However, ER sequestration may preserve the structural role of PltA in holotoxin assembly while limiting its contribution to intoxication, thereby reducing the overall potency of the toxin and promoting a host response that favors persistence rather than acute inflammatory disease.

### The *Salmonella* ADP‐ribosylating toxin AB (ArtAB) is not expressed by *S.* Typhi

The *artAB* operon is phage‐encoded and distributed amongst many *Salmonella* serovars [8, 29]. *S*. Typhi and Paratyphi A express ArtB (also called PltC in *S*. Typhi; *sty1364*), which can form a homopentamer [18, 30] or a heteropentamer with PltB [31, 32]. Both pentamers bind PltA/CdtB and can expand the range of cell types affected by typhoid toxin. However, the expression of a functional ArtAB toxin is limited to NTS because *S*. Typhi and Paratyphi A contain truncated *artA* pseudogenes. This was reported for NCBI sequences CP023975 and CP019185 [33], and we have since identified truncated *artA* pseudogenes in over 100 additional strains of *S*. Typhi and Paratyphi A. Others have also noted the presence of a degraded *artA* gene in *S*. Typhi [18, 30]. Previous studies reported an *artA* gene in *S*. Typhi and Paratyphi A, but the detection methods did not probe for full‐length *artA* [31, 34].

ArtA is 58% identical to PltA [33]. However, unlike PltA, ArtA contains an N‐terminal **RPPDVI** motif and exhibits cellular activity (Table 1) [29, 33, 35, 36]. *Escherichia coli* likewise produces a functional pertussis‐like toxin that contains a PltA subunit with an N‐terminal **RPPEEI** sequence (Table 1) [37]. These observations indicate the **RPP** motif of ArtA is responsible for its putative Hsp90‐dependent translocation to the cytosol and is thus indirectly responsible for the cytosolic activity of ArtA. Furthermore, the cellular activity of ArtA (**RPPDVI**) and lack of activity for *S*. Typhi PltA (**TPPDVI**) provides a “natural experiment” suggesting an **R**‐to‐**T** substitution in the PltA translocation motif prevents it from reaching the cytosol where it would elicit a cytopathic effect. The stealth infection facilitated by typhoid toxin thus appears to require attenuated ADPRT activity, either from toxin sequestration in the ER (PltA) or genetic ablation of the toxin catalytic subunit (ArtA). It should be noted that PltA cannot be ablated, as this would prevent the incorporation of CdtB into a typhoid holotoxin (Fig. 1).

### Potential impact of ADPRT activity on the immunosuppressive effects of typhoid toxin

The potent inflammatory effect of pertussis toxin is well‐characterized [38, 39, 40, 41, 42] and would be expected to be replicated by PltA in typhoid toxin. However, the initial stages of *S*. Typhi infection are usually asymptomatic due to suppression of the intestinal inflammatory response. This occurs despite the presence of PltA in typhoid toxin and eventually allows the bacterium to establish a persistent, systemic infection [5, 6, 7]. We propose that retention of PltA in the ER is necessary to prevent a strong inflammatory response which would impair early stealth invasion of the intestinal tract. We likewise hypothesize that an *artA* pseudogene is always present in the *S*. Typhi *artAB* locus because the expression of a functional ArtAB toxin would eliminate the anti‐inflammatory effects of CdtB and other *S*. Typhi virulence factors. Balanced pathogenicity – the evolutionary shift to a less virulent state that promotes pathogen carriage and shedding – may have thus eliminated the function of ADPRTs in *S*. Typhi.

The CdtB‐driven immunomodulatory effects of typhoid toxin are also seen in NTS that naturally express the toxin, as these serovars are more likely to be associated with invasive disease than *cdtB*‐negative NTS [43]. A mouse model of infection further indicated that NTS dissemination to extraintestinal sites involves the anti‐inflammatory action of typhoid toxin. The *S*. Javiana strain from this study encodes *artB* but has a truncated *artA* gene [12]. These observations are consistent with our model, which predicts NTS that express typhoid toxin will exhibit lower intestinal inflammation and a higher propensity for dissemination than NTS that express both typhoid toxin and ArtAB.

### Testing the model of balanced pathogenicity

We predict a **T**‐to‐**R** substitution in the native **TPPDVI** motif of PltA will promote Hsp90‐dependent PltA translocation to the cytosol, resulting in enhanced cellular toxicity and a strong inflammatory response that negates the initial asymptomatic infection of the intestinal tract. This hypothesis draws upon several published observations, but it has yet to be directly tested. As described below, our model could be interrogated using techniques in cell biology and physiological models of infection.

Studies using cells incubated with typhoid toxin or transfected with a PltA expression plasmid could definitively establish whether, as predicted, native **TPPDVI**‐PltA is retained in the ER while mutant **RPPDVI**‐PltA is exported from the ER to the cytosol. For this work, the intracellular distribution of PltA can be detected with antibody‐based methods after partitioning the cells into separate organelle and cytosol fractions [20, 44, 45]. Co‐immunoprecipitation, proximity ligation assay, or other methods could be used to determine whether, as predicted, Hsp90 binds to mutant **RPPDVI**‐PltA but not to native **TPPDVI**‐PltA. A role for Hsp90 in the predicted ER‐to‐cytosol export of **RPPDVI**‐PltA could be demonstrated by RNAi or drug treatment to inhibit Hsp90 function [20, 23]. Morphological and biochemical detection of toxin activity could further document the passage of PltA into the cytosol, as previously done for pertussis toxin [46, 47, 48]. Experiments with an intact typhoid toxin are important for this work because it is possible that additional mechanisms are responsible for the retention of PltA in the ER. For example, the continued association of PltA with PltB in the ER (i.e., lack of disassembly) would prevent holotoxin‐associated **RPPDVI**‐PltA but not transfected **RPPDVI**‐PltA from reaching the cytosol. Reduction of the CdtB/PltA disulfide bond is sufficient to release CdtB from typhoid toxin [3], so a stable PltA/PltB complex would not affect CdtB export from the ER. This collective approach has been used to study several ER‐translocating toxins [15, 19, 49, 50] and could be applied to PltA once the required reagents are available (recombinant constructs, purified PltA, and an antibody against PltA).

Using the *S*. Typhimurium MC1 strain that causes a systemic infection resembling typhoid fever in immunocompetent mice [9, 51, 52], it has been demonstrated that the expression of native typhoid toxin in strain MC1 (MC1 TT) promotes host survival and reduces the severity of intestinal inflammation through effects such as the presence of CD206 anti‐inflammatory macrophages and T regulatory lymphocytes, reduced recruitment of other leukocytes, and production of Th2‐type cytokines [9, 10, 11]. All effects were linked to the presence of CdtB. MC1 TT strains expressing a mutant **RPPDVI**‐PltA variant of typhoid toxin or co‐expressing native typhoid toxin with ArtAB could be used in this system to determine whether, as predicted, the immunosuppressive effects of typhoid toxin are minimized by the cytosolic activity of an ADPRT. These studies could provide further insight into *Salmonella* pathogenesis, including infections caused by NTS that naturally express both typhoid toxin and ArtAB.

The physical presence of PltA in the cytosol could trigger a signaling pathway that is sufficient to counteract the anti‐inflammatory properties of CdtB, which would explain why native PltA has a non‐functional **TPPDVI** translocation motif rather than an active site mutation. This possibility could be tested with a variant of PltA containing both the functional **RPPDVI** translocation motif and an E115A substitution that eliminates its ADP‐ribosyltransferase activity [4]. Cell‐based studies would confirm the delivery of this PltA variant to the cytosol and its lack of cytosolic activity, followed by an animal study to determine whether an MC1 TT strain expressing the PltA variant in typhoid toxin still supports anti‐inflammatory effects and host survival.

The physiological impact of an ADPRT on the anti‐inflammatory properties of typhoid toxin could also be examined with an *in vitro* human 3D “minigut” model that recapitulates the complexity of the intestinal mucosa [10, 53]. 3D miniguts are composed of immortalized, non‐transformed HCEC‐1CT human colonic epithelial cells and human colonic fibroblasts complemented with freshly isolated peripheral blood mononuclear cells (PBMCs). MC1 TT infection of the 3D miniguts does not induce destruction of the epithelial layer in the presence of PBMCs [10]. We therefore predict that miniguts exposed to MC1 TT strains expressing the **RPPDVI** variant of PltA or co‐expressing ArtAB with native typhoid toxin will generate a strong inflammatory environment associated with enhanced cellular toxicity and tissue destruction.

Ojiakor et al. [54] suggested an alternative explanation for the ablation of *artA* in *S*. Typhi and Paratyphi A genomes that contain *artB/pltC*: the absence of ArtA prevents it from competing with PltA for association with the cell‐binding moiety of typhoid toxin. With this model, the predicted inflammatory response resulting from ArtAB co‐expression with typhoid toxin could be attributed to the inefficient assembly of typhoid toxin in the presence of ArtA. To address this possibility, the amount of CdtB‐induced DNA damage caused by MC1 TT vs. MC1 TT co‐expressing ArtAB could be used as a proxy for the extent of typhoid toxin production in the two strains. Miniguts infected with MC1 TT could also be intoxicated with purified ArtAB, as the assembly of typhoid toxin would not be disrupted under this condition. We predict the anti‐inflammatory effects of typhoid toxin will be minimized by both co‐expression of typhoid toxin with ArtAB as well as typhoid toxin expression with exogenously applied ArtAB.

## Conclusion

Several pathogens produce a cytolethal distending toxin that facilitates long‐term colonization of the host through immunosuppressive effects [55]. The CdtB subunit of typhoid toxin appears to have a similar function that represses the intestinal inflammatory response, reduces the severity of infection, and allows *S*. Typhi to establish a chronic infection [9, 56]. PltA can ADP‐ribosylate an unidentified host protein from a cell extract, but it has no known activity against intact cells [3, 4]. There is currently no evidence for PltA translocation to the cytosol, yet there is a general assumption that this occurs and will be documented in future work [2, 3, 9, 17, 18]. We instead propose that PltA does not reach the cytosol, and we provide a molecular basis for its sequestration in the ER: PltA cannot access its cytosolic target because its **TPPDVI** sequence is not a substrate for Hsp90‐dependent export to the cytosol. Retention of PltA in the ER limits the potency of typhoid toxin, thereby facilitating an early‐stage asymptomatic infection. The strategy of ER sequestration for *S*. Typhi PltA is necessary because PltA must be expressed to form an intact typhoid holotoxin with the genotoxic activity of CdtB (Fig. 1). The absence of an intact *artA* gene in the *S*. Typhi genome likewise ensures that typhoid toxin can facilitate an initial stealth infection of the intestinal tract. This hypothesis represents a unique example of balanced pathogenicity in which attenuated ADPRT activity promotes increased bacterial fitness.

## Materials and methods

### Materials

Ham's F‐12 medium, Opti‐MEM, and Lipofectamine 3000 were purchased from Thermo Fisher Scientific (Waltham, MA, USA). Antibodies were purchased from Advanced Targeting Systems (CTA1; Carlsbad, CA, USA), Abcam (Hsp90 and Grp94; Cambridge, UK), or Jackson ImmunoResearch (secondary antibodies; West Grove, PA, USA). CHO K‐1 cells (catalog #CCL‐61, RRID:CVCL_0214) were obtained from the American Type Culture Collection (Manassas, VA) and maintained in Ham's F‐12 K medium (Gibco Life Technologies, Carlsbad, CA, USA) supplemented with 10% fetal bovine serum (R&D Systems, Minneapolis, MN, USA) and antibiotic‐antimycotic (Gibco Life Technologies).

### Translocation assay with the catalytic A1 subunit from cholera toxin (CTA1)

Chinese hamster ovary (CHO) cells were seeded in 6‐well plates and cultured overnight to reach approximately 80% confluence prior to transfection. On the day of transfection, cells were washed once with phosphate‐buffered saline (PBS) before 900 μL of Opti‐MEM medium was added to each well. Lipofectamine 3000 was diluted 1 : 12.33 in Opti‐MEM and incubated at room temperature for 10 min. In parallel, plasmid DNA encoding ER‐localized variants of either wild‐type **RPPDEI**‐CTA1 or mutant **TPPDEI**‐CTA1 [20] was diluted in Opti‐MEM to a final quantity of 2 μg per condition and incubated at room temperature for 10 min. The diluted DNA and Lipofectamine 3000 solutions were then combined at a 1 : 1 ratio and incubated for an additional 10 min at room temperature. A total of 100 μL of the transfection mixture was added to each corresponding well and incubated for 4 h at 37 °C. The cells were then washed once with PBS before adding fresh serum‐ and antibiotic‐free F‐12 medium for overnight incubation.

Following the transfection, cells were washed twice with PBS, trypsinized for 5 min at 37 °C, collected, and centrifuged at 100× ***g*** for 5 min at 4 °C. Cell pellets were incubated on ice for 10 min, then permeabilized with 25 μg·mL^−1^ of digitonin in 100 μL ice‐cold HCN buffer (50 mm HEPES, pH 7.5; 150 mm NaCl; 2 mm CaCl_2_; 10 mm N‐ethylmaleimide; and protease inhibitor cocktail) for an additional 10 min on ice. After permeabilization, cells were centrifuged at 2000× ***g*** for 5 min at 4 °C to isolate the cytosolic fraction (supernatant), which was transferred to a fresh tube and supplemented with 20 μL of 4X sample buffer. The remaining pellet fraction that contained intact organelles was resuspended in 120 μL of 1X sample buffer. Although all endomembrane organelles are present in the pellet, previous microscopy and biochemical methods have shown that CTA1 and other toxin A chains are initially located in the ER when transfected into mammalian cells with an N‐terminal signal sequence [46, 57, 58, 59, 60].

Samples were resolved by SDS/PAGE using 15% polyacrylamide gels and transferred onto polyvinylidene difluoride membranes using a semidry transfer system (Bio‐Rad, Hercules, CA, USA). Membranes were blocked for 30 min at room temperature with 4% non‐fat milk in TBST (25 mm Tris/HCl, pH 8.0; 125 mm NaCl; 0.1% Tween‐20), followed by overnight incubation at 4 °C with a rabbit anti‐CTA1 antibody (1 : 5000 in 4% milk‐TBST). The membrane was then washed four times (5 min each) with TBST and incubated with a goat anti‐rabbit IgG secondary antibody conjugated to horseradish peroxidase (1 : 20 000 in 4% milk‐TBST) for 30 min. The membrane was then washed again as previously described and developed using a chemiluminescent detection solution composed of 100 mm Tris/HCl (pH 8.8), 1.25 mm luminol, 0.225 mm coumaric acid, and 3.04 mm hydrogen peroxide.

To confirm the fidelity of cellular fractionation, both the pellet and supernatant fractions from each transfection were resolved by SDS/PAGE and transferred to polyvinylidene difluoride membranes. These membranes were blocked for 30 min at room temperature in 4% milk‐TBST and incubated overnight at 4 °C with a cocktail of mouse anti‐Hsp90 antibody and rabbit anti‐GRP94 antibody (both at 1 : 15 000 in 4% milk‐TBST). Hsp90 was used as a marker for the cytosol, while Grp94 was used as a marker for the ER lumen. After washing as described above, membranes were incubated for 30 min at room temperature with goat anti‐mouse IgG and goat anti‐rabbit IgG secondary antibodies conjugated to horseradish peroxidase (1 : 20 000 in 4% milk‐TBST) before application of the chemiluminescent detection solution.

### Identification of the *artAB* locus in *S.* Typhi and Paratyphi A

The *artAB* operon and the homologous *pltC* region were identified in *S*. Typhi and *S*. Paratyphi A serovars using whole genome sequences available from the National Center for Biotechnology Information NCBI. The *artAB* operon from *S*. Typhimurium DT104 [29] was used as the query sequence for nucleotide BLAST to search *S*. Typhi (TAXID 90370) and *S*. Paratyphi A (TAXID 54388) with a maximum of 1000 target sequences. Retrieved protein sequences were aligned and evolutionary analysis completed using mega 7 [61]. A rooted dendrogram of exhaustive pairwise AB_5_ toxin alignments of 20 sequences (A subunits) or 19 sequences (B subunits) was constructed using the maximum‐likelihood method based on the JTT matrix‐based model [33].

## Conflict of interest

The authors declare there are no conflicts of interest.

## Author contributions

JKT, TF, TA, KT contributed to conceptualization; MCZ‐R, JKT, TF, TA, KT contributed to data analysis; JKT, MCZ‐R contributed to investigation; KT contributed to supervision; JKT, TF, TA, KT contributed to writing—original draft; MCZ‐R, JKT, TF, TA, KT contributed to writing—review and editing.

## Data acessibility

Data supporting the findings of this study are available in the main figures of the article. Original experiments are available from the corresponding author (kteter@mail.ucf.edu) upon reasonable request.
